# Supplementary material for: Qtlizer: comprehensive QTL annotation of GWAS results
Source: Sci Rep. 2020 Nov 24;10:20417. doi: 10.1038/s41598-020-75770-7 (PMC7687904; doi:10.1038/s41598-020-75770-7)
Supplement: Supplementary file 2 — Supplementary Information 2. [file 41598_2020_75770_MOESM2_ESM.pdf]

Supplementary Material

Qtlizer: comprehensive QTL annotation of GWAS results

Matthias Munz[1-5,*], Inken Wohlers[3], Eric Simon[6], Tobias Reinberger[1,4-5], Hauke Busch[3], Arne S. Schaefer[2,¶], Jeanette Erdmann[1,4-5,¶,*]

[¶]These authors contributed equally

[1]Institute for Cardiogenetics, University of Lübeck, 23562 Lübeck, Germany
[2]Charité – University Medicine Berlin, corporate member of Freie Universität Berlin, Humboldt-Universität zu Berlin, and Berlin Institute of Health, Institute for Dental and Craniofacial Sciences, Department of Periodontology and Synoptic Dentistry, 14197 Berlin, Germany
[3]Medical Systems Biology Group, Institute of Experimental Dermatology, Institute for Cardiogenetics, University of Lübeck, 23562 Lübeck, Germany
[4]DZHK (German Research Centre for Cardiovascular Research), partner site Hamburg/Lübeck/Kiel, 23562 Lübeck, Germany
[5]University Heart Center Lübeck, 23562 Lübeck, Germany
[6]Computational Biology, Boehringer Ingelheim Pharma GmbH & Co KG, 88397 Biberach, Germany

[*] To whom correspondence should be addressed.
Email: m.munz@uni-luebeck.de
Telephone: +49 (0) 451 500 50860
Address:
Medical Systems Biology Group
Institute of Experimental Dermatology
Institute for Cardiogenetics
University of Lübeck
Ratzeburger Allee 160
23562 Lübeck, Germany

Correspondence may also be addressed to
Email: jeanette.erdmann@uni-luebeck.de
Telephone: +49 (0) 451 3101 8300
Address:
Institute for Cardiogenetics
University of Lübeck
Ratzeburger Allee 160
23562 Lübeck, Germany

Supplementary Figure 1. Linkage disequilibrium (LD) was calculated with PLINK 1.9 beta (https://www.cog-genomics.org/plink2/). The script shows the parameters that we passed to PLINK for calculating LD. Among these, `--ld-window` sets the maximum window size as number of variants, whereas `--ld-window-kb` sets the maximum window size in kilo bases (kb). PLINK considers the window size which is reached first. Given a variant, the window size determines for how many downstream (regarding chromosomal position) variants the LD should be calculated for a given variant.. 4

Supplementary Figure 2. Regular expressions that are used by Qtlizer to extract (a) rsIDs, (b) chromosomal positions and (c) gene identifier. These expressions are applied in the backend (Java) and frontend (Javascript) of Qtlizer. Regarding Javascript interpretation, it has to be stated that, to-date, lookbehind assertions `?<=` are only be supported by the Google Chrome browser, but not by Firefox, Safari and Edge. Therefore, we converted the regexes accordingly (d-f).. 5

Supplementary Figure 3. Regional association plot showing an association signal on chromosome 3 at the gene locus MRAS. Each dot represents a single nucleotide polymorphism (SNP). The colour coding reflects the degree of linkage disequilibrium (r^2) between a variant and the index variant rs2306374. This plot visualizes GWAS data on coronary artery disease provided by the CARDIoGRAMplusC4D Consortium (http://www.cardiogramplusc4d.org/data-downloads/). 6

Supplementary Figure 4. For larger queries Qtlizer can be accessed using the Genehopper REST API. The script describes an example request in **R**. More info about the API can be found at http://www.genehopper.de/rest. 7

Supplementary Figure 5. The script describes an example request in **Perl** (http://www.genehopper.de/rest). 8

Supplementary Table 1. List of QTL datasets that were integrated into Qtlizer....................................... 9

Supplementary Table 2. For each of the investigated LD thresholds (r^2) top 100 genes with highest number of datasets per eQTL (n_max), annotated by mean expression percentile in GTEx (mean_expr_percentile) and by housekeeping gene status (is_housekeeping). Table is available in attached Excel sheet. 12

Supplementary Table 3. For each LD threshold the number of housekeeping genes and the number of genes in the 10% highest expressed genes in GTEx v8 among the 100 genes with the highest dataset count per eQTL LD block. Adjusted (FDR < 0.05) P-values were calculated using hypergeometric test assuming an overall gene number of 18,521 (genes with at least one best significant eQTL), a total of 3,705 housekeeping genes and a total of 1,201 genes among the 10% highest expressed genes............... 13

Supplementary Table 4. Members of the Cardiogenics Consortium. 14

```
#!/bin/bash


/path/to/plink --bfile <bim/bed/fam prefix> --r2 --ld-window 99999 --ld-window-kb
1000 --ld-window-r2 0.2 --out <output_file>
```

Supplementary Figure 1. Linkage disequilibrium (LD) was calculated with PLINK 1.9 beta (https://www.cog-genomics.org/plink2/). The script shows the parameters that we passed to PLINK for calculating LD. Among these, `--ld-window` sets the maximum window size as number of variants, whereas `--ld-window-kb` sets the maximum window size in kilo bases (kb). PLINK considers the window size which is reached first. Given a variant, the window size determines for how many downstream (regarding chromosomal position) variants the LD should be calculated for a given variant.

```
a)

"(?<=^|[\\s,;])rs(\\d+){1,12}(?=[\\s,;]|$)"

b)

"(?<=^|[\\s,;])(hg19|hg38|grch37|grch38)[:_-](chr)?(1[0-9]|2[0-2]|[1-
9]|x|y|mt)[:_-](\\d+){1,8}(?=[\\s,;:_-]|$|[atgc]+)"

c)

"(?<=^|[\\s,;])([a-z0-9]+){2,20}(?=[\\s,;]|$)"

d)

Array.from(new Set(q.match(/(^|[\s,;])rs(\d+){1,12}(?=[\s,;]|$)/g)))
      .map(function(e) {return e.replace(/^[\s,;]/g, "")});

e)

Array.from(new Set(q.match(/(^|[\s,;])(hg19|hg38|grch37|grch38)[:_-](chr)?(1[0-
9]|2[0-2]|[1-9]|x|y|mt)[:_-](\d+){1,8}(?=[\s,;:_-]|$|[atgc]+)/g)))
      .map(function(e) {return e.replace(/^[\s,;]/g, "")
                               .replace(/_|-/g, ":")
                               .replace(/grch37/g, "hg19")
                               .replace(/grch38/g, "hg38")
                               .replace(/chr/g, "");});

f)

Array.from(new Set(q.match(/(^|[\s,;])([a-z0-9]+){2,20}(?=[\s,;]|$)/g)))
      .map(function(e) {return e.replace(/^[\s,;]/g, "")})
                               .filter(function(v){return !v.match(/^rs\d+$/)});
```

Supplementary Figure 2. Regular expressions that are used by Qtlizer to extract (a) rsIDs, (b) chromosomal positions and (c) gene identifier. These expressions are applied in the backend (Java) and frontend (Javascript) of Qtlizer. Regarding the Javascript implementation, lookbehind assertions `?<=` have only recently been supported by all common browsers. To ensure backwards compatibility, we converted the regexes accordingly (d-f).

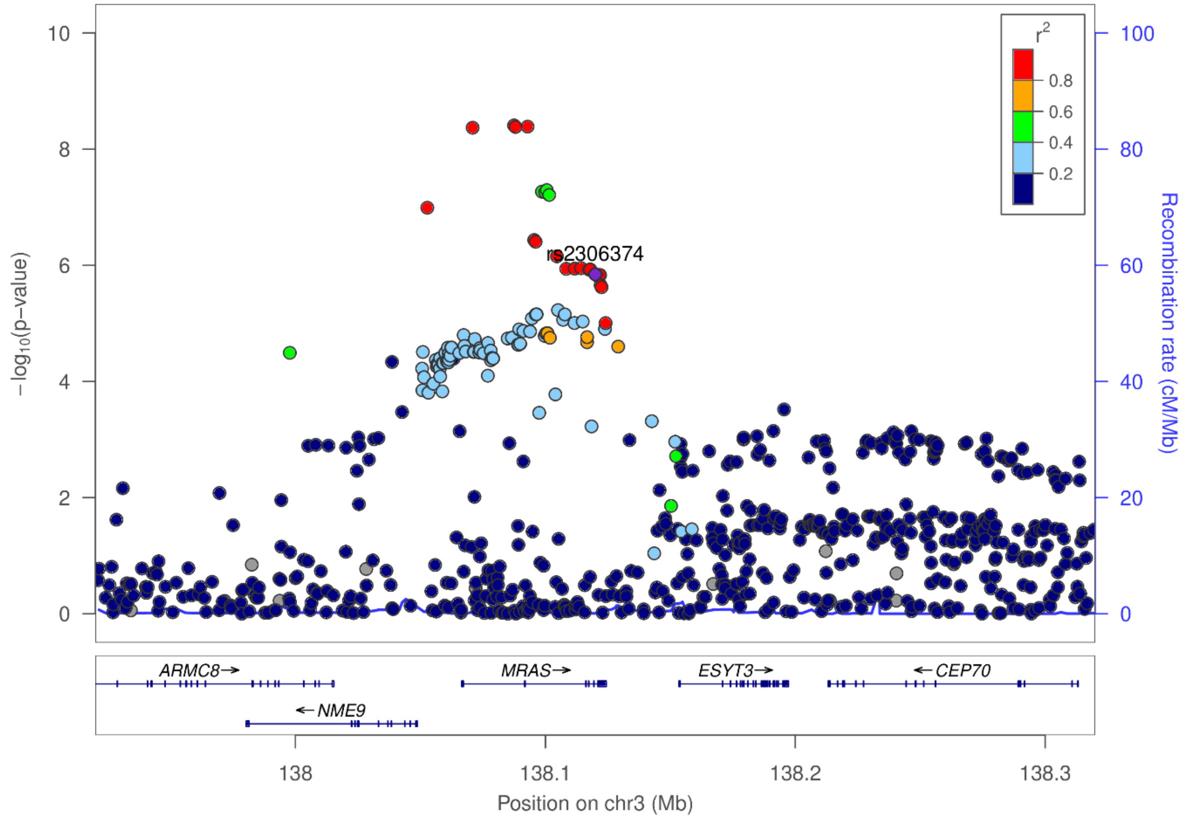

Supplementary Figure 3. Regional association plot showing an association signal on chromosome 3 at the gene locus *MRAS*. Each dot represents a single nucleotide polymorphism (SNP). The colour coding reflects the degree of linkage disequilibrium (r^2) between a variant and the index variant rs2306374. This plot visualizes GWAS data on coronary artery disease provided by the CARDIoGRAMplusC4D Consortium (http://www.cardiogramplusc4d.org/data-downloads/).

```
# https://cran.r-project.org/web/packages/httr/vignettes/quickstart.html
library(httr)

q = "rs4284742,rs2070901,rs123"
corr = 0.8 # optional
ld_method = "r2" # optional

url = paste('http://www.genehopper.de/rest/qtlizer?q=', q, "&corr=", corr,
"&ld_method=", ld_method, sep="")

response = POST(url);
result = content(response)

a = unlist(strsplit(result , "\n|\t"))
m = matrix(a[20:length(a)], ncol=19, byrow=TRUE)
d = as.data.frame(m, stringsAsFactors=FALSE)
colnames(d) = a[1:19]

write.table(d, file="example.txt", sep="\t", quote = FALSE, append=FALSE,
row.names = FALSE, col.names = TRUE)
```

Supplementary Figure 4. For larger queries Qtlizer can be accessed using the Genehopper REST API. The script describes an example request in **R**. More info about the API can be found at http://www.genehopper.de/rest.

```
#!/usr/bin/perl

use warnings;
use strict;
use LWP::UserAgent;

my $url = 'http://www.genehopper.de/rest/qtlizer';

my %parameters = (
        "q" =>  "rs4284742, rs2070901, rs123",
        "corr" => 0.8, # optional
        "ld_method" => "r2" # optional
);

my $url_with_pars = "$url?";
$url_with_pars .= $_."=".$parameters{$_}."&" for keys(%parameters);


my $ua = LWP::UserAgent->new();
my $response = $ua -> post($url_with_pars);
my $content = $response -> decoded_content();

print $content."\n";
```

Supplementary Figure 5. The script describes an example request in **Perl** (http://www.genehopper.de/rest).

Supplementary Table 1. List of QTL datasets that were integrated into Qtlizer.

Source	PMID	Tissue	QTL Type	#Variants
Blood eQTL Browser	24013639	Peripheral blood	eQTL	768850
Franzén et al. (Science 2016)	27540175	Adipose - Subcutaneous	eQTL	6776
Franzén et al. (Science 2016)	27540175	Adipose - Visceral (Abdominal)	eQTL	6697
Franzén et al. (Science 2016)	27540175	Adrenal gland	eQTL	1
Franzén et al. (Science 2016)	27540175	Artery - Aorta	eQTL	288
Franzén et al. (Science 2016)	27540175	Artery - Mammary	eQTL	7109
Franzén et al. (Science 2016)	27540175	Artery - Tibial	eQTL	12
Franzén et al. (Science 2016)	27540175	Atherosclerotic aortic root	eQTL	6958
Franzén et al. (Science 2016)	27540175	Blood	eQTL	5591
Franzén et al. (Science 2016)	27540175	Brain - Hypothalamus	eQTL	2
Franzén et al. (Science 2016)	27540175	Brain - Nucleus accumbens (Basal ganglia)	eQTL	2
Franzén et al. (Science 2016)	27540175	Brain - Putamen (Basal ganglia)	eQTL	11
Franzén et al. (Science 2016)	27540175	Cells - Transformed fibroblasts	eQTL	658
Franzén et al. (Science 2016)	27540175	Colon - Transverse	eQTL	18
Franzén et al. (Science 2016)	27540175	Esophagus - Mucosa	eQTL	976
Franzén et al. (Science 2016)	27540175	Esophagus - Muscularis	eQTL	15
Franzén et al. (Science 2016)	27540175	Heart - Left ventricle	eQTL	3
Franzén et al. (Science 2016)	27540175	Liver	eQTL	7043
Franzén et al. (Science 2016)	27540175	Lung	eQTL	98
Franzén et al. (Science 2016)	27540175	Muscle skeletal	eQTL	5650
Franzén et al. (Science 2016)	27540175	Nerve - Tibial	eQTL	30
Franzén et al. (Science 2016)	27540175	Pancreas	eQTL	283
Franzén et al. (Science 2016)	27540175	Prostate	eQTL	1
Franzén et al. (Science 2016)	27540175	Skin - Not sun exposed (Suprapubic)	eQTL	11
Franzén et al. (Science 2016)	27540175	Skin - Sun exposed (Lower leg)	eQTL	64
Franzén et al. (Science 2016)	27540175	Testis	eQTL	203
Franzén et al. (Science 2016)	27540175	Thyroid	eQTL	2120
Franzén et al. (Science 2016)	27540175	Whole blood	eQTL	35
GEUVADIS	24037378	Cells - Lymphoblastoid cell lines	eQTL	404608
GRASP 2 Catalog	17873877	Cells - Lymphoblastoid cell lines	eQTL	8417
GRASP 2 Catalog	18193047	Cells - Lymphoblastoid cell lines	eQTL	463
GRASP 2 Catalog	18478092	Cells - Leukemia cells	eQTL	6
GRASP 2 Catalog	18478092	Cells - Normal peripheral leukocytes	eQTL	6
GRASP 2 Catalog	19128478	Blood cells in celiac disease	eQTL	2812
GRASP 2 Catalog	19222302	Brain - Cortex	eQTL	1281
GRASP 2 Catalog	19222302	Cells - Peripheral blood mononuclear cells	eQTL	2420
GRASP 2 Catalog	19361613	Brain - Cortex with alzheimer's interaction	eQTL	252
GRASP 2 Catalog	19361613	Brain - Cortex with no alzheimer's interaction	eQTL	1117
GRASP 2 Catalog	19644074	Cells - Fibroblasts	eQTL	512
GRASP 2 Catalog	19644074	Cells - Lymphoblastoid cell lines	eQTL	551
GRASP 2 Catalog	19644074	Cells - T cells	eQTL	532
GRASP 2 Catalog	19654370	Osteoblasts	eQTL	711
GRASP 2 Catalog	19680542	Cells - Lymphoblastoid cell lines	eQTL	73
GRASP 2 Catalog	19680542	Osteoblasts	eQTL	133
GRASP 2 Catalog	19966804	Cells - Peripheral leukocytes	eQTL	215
GRASP 2 Catalog	20084173	Liver	eQTL	49
GRASP 2 Catalog	20170901	Cells - Aortic endothelial cells	eQTL	5
GRASP 2 Catalog	20170901	Cells - Aortic endothelial cells treated with OX-PAPC	eQTL	35
GRASP 2 Catalog	20351726	Brain - Prefrontal cortex	eQTL	3035
GRASP 2 Catalog	20833654	Cells - CD4+ lymphocytes	eQTL	6843
GRASP 2 Catalog	20844574	Blood	eQTL	9
GRASP 2 Catalog	21226949	Endometrial tumors	eQTL	229
GRASP 2 Catalog	21637794	Liver	eQTL	7970
GRASP 2 Catalog	21789213	Cells - Lymphoblastoid cell lines	eQTL	8
GRASP 2 Catalog	21829388	Adipose - Subcutaneous (Abdominal)	eQTL	3
GRASP 2 Catalog	21829388	Adipose - Visceral (Omentum majus)	eQTL	6
GRASP 2 Catalog	21829388	Blood	eQTL	86912
GRASP 2 Catalog	21829388	Musculus rectus - Abdominis	eQTL	3
GRASP 2 Catalog	21949713	Sputum	eQTL	2047
GRASP 2 Catalog	22006096	Liver	eQTL	1376
GRASP 2 Catalog	22233810	Cells - Dendritic cells	eQTL	9434
GRASP 2 Catalog	22233810	Cells - Dendritic cells treated with mycobacterium tuberculosis	eQTL	9488
GRASP 2 Catalog	22383892	Adipose - Gluteal	eQTL	11
GRASP 2 Catalog	22383892	Adipose - Subcutaneous (Abdominal)	eQTL	30
GRASP 2 Catalog	22447449	Liver and corresponding expression modules	eQTL	28
GRASP 2 Catalog	22447449	Liver	eQTL	1
GRASP 2 Catalog	22509407	Whole blood in amyotrophic lateral sclerosis cases and controls	eQTL	15

GRASP 2 Catalog	22522925	Breast tumors	eQTL	2981
GRASP 2 Catalog	22685416	Brain - Cerebellum in alzheimer's disease cases and controls	eQTL	20554
GRASP 2 Catalog	22685416	Brain - Cerebellum in alzheimer's disease cases	eQTL	3541
GRASP 2 Catalog	22685416	Brain - Cerebellum in non-alzheimer's disease samples	eQTL	3107
GRASP 2 Catalog	22685416	Brain - Cerebellum in progressive supranuclear palsy cases	eQTL	1610
GRASP 2 Catalog	22685416	Brain - Temporal cortex in alzheimer's disease cases and controls	eQTL	20069
GRASP 2 Catalog	22685416	Brain - Temporal cortex in alzheimer's disease cases	eQTL	3204
GRASP 2 Catalog	22685416	Brain - Temporal cortex in non-alzheimer's disease samples	eQTL	1747
GRASP 2 Catalog	22685416	Brain - Temporal cortex in progressive supranuclear palsy cases	eQTL	1117
GRASP 2 Catalog	22692066	Whole blood	eQTL	1470
GRASP 2 Catalog	22912676	Cells - B-lymphoblastoid cell lines	eQTL	25
GRASP 2 Catalog	23474282	Ileum - Pre-pouch	eQTL	71037
GRASP 2 Catalog	23671422	Cells - Fibroblasts	eQTL	1
GRASP 2 Catalog	23671422	Cells - Fibroblasts, T cells and B-lymphoblastoid cell lines	eQTL	2
GTEx v7	23715323	Adipose - Subcutaneous	eQTL	1492748
GTEx v7	23715323	Adipose - Visceral (Omentum)	eQTL	989804
GTEx v7	23715323	Adrenal gland	eQTL	521055
GTEx v7	23715323	Artery - Aorta	eQTL	1034149
GTEx v7	23715323	Artery - Coronary	eQTL	338471
GTEx v7	23715323	Artery - Tibial	eQTL	1547788
GTEx v7	23715323	Brain - Amygdala	eQTL	150681
GTEx v7	23715323	Brain - Anterior cingulate cortex (BA24)	eQTL	276912
GTEx v7	23715323	Brain - Caudate (Basal ganglia)	eQTL	414047
GTEx v7	23715323	Brain - Cerebellar hemisphere	eQTL	543823
GTEx v7	23715323	Brain - Cerebellum	eQTL	780268
GTEx v7	23715323	Brain - Cortex	eQTL	455391
GTEx v7	23715323	Brain - Frontal cortex (BA9)	eQTL	327068
GTEx v7	23715323	Brain - Hippocampus	eQTL	211589
GTEx v7	23715323	Brain - Hypothalamus	eQTL	214764
GTEx v7	23715323	Brain - Nucleus accumbens (Basal ganglia)	eQTL	357772
GTEx v7	23715323	Brain - Putamen (Basal ganglia)	eQTL	278404
GTEx v7	23715323	Brain - Spinal cord (Cervical c-1)	eQTL	167028
GTEx v7	23715323	Brain - Substantia nigra	eQTL	112251
GTEx v7	23715323	Breast - Mammary tissue	eQTL	695994
GTEx v7	23715323	Cells - EBV-transformed lymphocytes	eQTL	271398
GTEx v7	23715323	Cells - Transformed fibroblasts	eQTL	1278165
GTEx v7	23715323	Colon - Sigmoid	eQTL	661130
GTEx v7	23715323	Colon - Transverse	eQTL	784629
GTEx v7	23715323	Esophagus - Gastroesophageal junction	eQTL	697016
GTEx v7	23715323	Esophagus - Mucosa	eQTL	1474044
GTEx v7	23715323	Esophagus - Muscularis	eQTL	1384504
GTEx v7	23715323	Heart - Atrial appendage	eQTL	841069
GTEx v7	23715323	Heart - Left ventricle	eQTL	740015
GTEx v7	23715323	Liver	eQTL	305452
GTEx v7	23715323	Lung	eQTL	1385916
GTEx v7	23715323	Minor salivary gland	eQTL	125852
GTEx v7	23715323	Muscle skeletal	eQTL	1438236
GTEx v7	23715323	Nerve - Tibial	eQTL	1837615
GTEx v7	23715323	Ovary	eQTL	262592
GTEx v7	23715323	Pancreas	eQTL	696296
GTEx v7	23715323	Pituitary	eQTL	552811
GTEx v7	23715323	Prostate	eQTL	283528
GTEx v7	23715323	Skin - Not sun exposed (Suprapubic)	eQTL	1250843
GTEx v7	23715323	Skin - Sun exposed (Lower leg)	eQTL	1697927
GTEx v7	23715323	Small intestine - Terminal ileum	eQTL	302654
GTEx v7	23715323	Spleen	eQTL	494717
GTEx v7	23715323	Stomach	eQTL	596021
GTEx v7	23715323	Testis	eQTL	1417129
GTEx v7	23715323	Thyroid	eQTL	2017804
GTEx v7	23715323	Uterus	eQTL	169796
GTEx v7	23715323	Vagina	eQTL	167241
GTEx v7	23715323	Whole blood	eQTL	1001052
Haploreg v4.1	17873874	Cells - Lymphoblastoid cell lines	eQTL	9979
Haploreg v4.1	18462017	Liver	eQTL	3603
Haploreg v4.1	20220756	Cells - Lymphoblastoid cell lines	eQTL	2049
Haploreg v4.1	20485568	Brain - Cerebellum	eQTL	5233
Haploreg v4.1	20485568	Brain - Frontal cortex	eQTL	5422
Haploreg v4.1	20485568	Brain - Pons	eQTL	3377
Haploreg v4.1	20485568	Brain - Temporal cortex	eQTL	5204
Haploreg v4.1	21283786	Osteoblasts BMP2	eQTL	1533

Haploreg v4.1	21283786	Osteoblasts DEX	eQTL	1248
Haploreg v4.1	21283786	Osteoblasts	eQTL	1089
Haploreg v4.1	21283786	Osteoblasts PGE2	eQTL	1519
Haploreg v4.1	23209423	Lung	eQTL	14977
Haploreg v4.1	24604202	Cells - Monocytes	eQTL	5982
Haploreg v4.1	24604202	Cells - Monocytes IFN	eQTL	9570
Haploreg v4.1	24604202	Cells - Monocytes LPS24	eQTL	2458
Haploreg v4.1	24604202	Cells - Monocytes LPS2	eQTL	3864
Haploreg v4.1	24846176	Heart	eQTL	5627
Haploreg v4.1	25174004	Brain - Aveall	eQTL	10597
Haploreg v4.1	25174004	Brain - Cerebellum	eQTL	2094
Haploreg v4.1	25174004	Brain - Frontal cortex	eQTL	1200
Haploreg v4.1	25174004	Brain - Hippocampus	eQTL	1003
Haploreg v4.1	25174004	Brain - Inferior olivary nucleus	eQTL	831
Haploreg v4.1	25174004	Brain - Intralobular white matter	eQTL	1515
Haploreg v4.1	25174004	Brain - Occipital cortex	eQTL	954
Haploreg v4.1	25174004	Brain - Putamen	eQTL	550
Haploreg v4.1	25174004	Brain - Substantia nigra	eQTL	415
Haploreg v4.1	25174004	Brain - Temporal Cortex	eQTL	1391
Haploreg v4.1	25174004	Brain - Thalamus	eQTL	841
Kim et al. (Nature Comm 2017)	28814792	Cells - Monocytes	eQTL	2403
Kim et al. (Nature Comm 2017)	28814792	Cells - Monocytes (stimulated with LPS/IVT/MDP vs. unstimulated)	reQTL	369
pGWAS	28240269	Blood plasma	pQTL	508
ScanDB	19933162	Brain - Cerebellum	eQTL	621247
ScanDB	19933162	Brain - Parietal lobe	eQTL	592490
ScanDB	19933162	Liver	eQTL	285990
seeQTL	17982457	Brain	eQTL	39154
seeQTL	22171328	Cells - Lymphoblastoid cell lines	eQTL	123727
The Cardiogenics Project	23382694	Cells - Monocytes	eQTL	1329875
The Cardiogenics Project	27558669	Cells - Macrophages	eQTL	1032604
Zeller et al. (PlosONE 2010)	20502693	Cells - Monocytes	eQTL	203642

Supplementary Table 2. For each of the investigated LD thresholds (r^2) top 100 genes with highest number of datasets per eQTL (n_max), annotated by mean expression percentile in GTEx (mean_expr_percentile) and by housekeeping gene status (is_housekeeping). Table is available in attached Excel sheet.

Supplementary Table 3. For each LD threshold the number of housekeeping genes and the number of genes in the 10% highest expressed genes in GTEx v8 among the 100 genes with the highest dataset count per eQTL LD block. Adjusted (FDR < 0.05) P-values were calculated using hypergeometric test assuming an overall gene number of 18,521 (genes with at least one best significant eQTL), a total of 3,705 housekeeping genes and a total of 1,201 genes among the 10% highest expressed genes.

LD threshold	#Housekeeping	Adjusted P	90th percentile expression	Adjusted P
No LD	29	1.84E-02	9	0.20
r2 = 1	33	1.65E-03	12	0.06
r2 > 0.95	42	2.12E-06	14	0.03
r2 > 0.9	41	3.12E-06	12	0.06
r2 > 0.8	37	1.05E-04	10	0.17
r2 > 0.6	36	1.89E-04	9	0.20

Supplementary Table 4. Members of the Cardiogenics Consortium.

Tony Attwood	Seraya Maouche
Stephanie Belz	Jasbir S Moore
Peter Braund	Gilles Montalescot
Jessy Brocheton	David Muir
François Cambien	Elizabeth Murray
Jason Cooper	Chris P Nelson
Abi Crisp-Hihn	Jessica Neudert
Panos Deloukas	David Niblett
Patrick Diemert	Karen O'Leary
Jeannette Erdmann	Willem H. Ouwehand
Nicola Foad	Helen Pollard
Tiphaine Godefroy	Carole Proust
Alison H. Goodall	Angela Rankin
Jay Gracey	Augusto Rendon
Emma Gray	Catherine M Rice
Rhian Gwilliams	Hendrik Sager
Susanne Heimerl	Nilesh J. Samani
Christian Hengstenberg	Jennifer Sambrook
Jennifer Jolley	Gerd Schmitz
Unni Krishnan	Michael Scholz
Heather Lloyd-Jones	Laura Schroeder
Ulrika Liljedahl	Heribert Schunkert
Ingrid Lugauer	Jonathan Stephens
Per Lundmark	Ann-Christine Syvannen
Chris Wallace	Stefanie Tennstedt
